# Case Report: Spontaneous Postpartum Quadruple Cervicocephalic Arterial Dissection With a Heterozygous *COL5A1* Variant of Unknown Significance

**DOI:** 10.3389/fneur.2022.928803

**Published:** 2022-07-12

**Authors:** Takeaki Imamura, Takaki Omura, Nobuo Sasaki, Satoshi Arino, Haruna Nohara, Akira Saito, Maki Ichinose, Kazumasa Yamaguchi, Naoki Kojima, Hiroshi Inagawa, Katsutoshi Takahashi, Toshiyuki Unno, Hiroko Morisaki, Osamu Ishikawa, Gakushi Yoshikawa, Yasusei Okada

**Affiliations:** ^1^Department of Emergency and Critical Care Medicine, Showa General Hospital, Tokyo, Japan; ^2^Department of Virology, Tohoku University Graduate School of Medicine, Sendai, Japan; ^3^Department of Neurosurgery, The University of Tokyo, Tokyo, Japan; ^4^Department of Neurosurgery, Saitama Medical Centre, Saitama, Japan; ^5^Department of Anesthesiology, Showa General Hospital, Tokyo, Japan; ^6^Department of Intensive Care, Toranomon Hospital, Tokyo, Japan; ^7^Department of Metabolism, Showa General Hospital, Tokyo, Japan; ^8^Department of Radiology, Showa General Hospital, Tokyo, Japan; ^9^Department of Medical Genetics, Sakakibara Heart Institute, Tokyo, Japan; ^10^Department of Neurosurgery, Asama General Hospital, Nagano, Japan; ^11^Department of Neurosurgery, Showa General Hospital, Tokyo, Japan

**Keywords:** postpartum, pregnancy, cervicocephalic arterial dissection, quadruple, *COL5A1* gene mutation

## Abstract

Pregnancy-associated cervicocephalic arterial dissection is rare, and its pathophysiology remains poorly understood. Despite the hypothesized contribution to pathogenesis, connective tissue diseases and genetic factors are rarely identified in clinical cases. We describe a case of postpartum arterial dissection involving all four cervicocephalic arteries resulting in acute cerebral infarction. The patient underwent successful endovascular thrombectomy and angioplasty and recovered fully without sequelae. Genetic screening for connective tissue diseases identified a heterozygous missense *COL5A1* variant with unknown clinical significance. Two genetically related family members later developed arterial abnormalities, and one of them tested positive for the same *COL5A1* gene variant as our patient, while the other was scheduled for genetic testing. The extensive clinical presentation of our patient and the prevalence of arterial abnormalities in her family warrant further assessment of the association between the identified *COL5A1* gene variant and the pathogenesis of arterial dissections.

## Introduction

Pregnancy-associated arterial dissection is estimated to occur in 0.006% of hospitalized pregnant or postpartum women. Of these, 61.5% are detected during the postpartum period, and dissected lesions involve the coronary (38.2%), vertebral (22.9%), aortic (19.8%), and carotid (19.5%) arteries (1). Previous studies revealed the association between pregnancy and cervicocephalic arterial dissection (CCAD), but its pathophysiology remains poorly understood (2). Proposed mechanisms include hemodynamic stress-induced disruption of vessel wall integrity, coagulative changes, and environmental and genetic factors (2, 3). It is hypothesized that genetic factors constitute a part of a multifactorial predisposition to CCAD (4). However, the identification of genetic factors related to connective tissue diseases is rare among CCAD cases, even in the extensive presentation of triple or quadruple CCAD regardless of pregnancy (4–6). Five cases of quadruple CCAD during pregnancy and puerperium have been reported in the literature, but no connective tissue diseases and genetic factors were mentioned or identified in those reports (7–11). We present a case of postpartum arterial dissection involving all four cervicocephalic arteries resulting in acute cerebral infarction. The patient underwent thrombectomy and angioplasty following the administration of recombinant tissue plasminogen activator (rt-PA) and recovered fully without neurological sequelae. Genetic screening for connective tissue diseases identified a heterozygous missense collagen type V alpha 1 chain (*COL5A1*) variant with unknown clinical significance.

## Case Description

A 37-year-old Caucasian female presented to our emergency department (ED) with a sudden onset of right facial paresis, right hemiparesis, and motor aphasia; her initial National Institutes of Health Stroke Scale (NIHSS) score was 12. The patient had given birth to her first child *via* vaginal delivery under epidural analgesia 10 days before presenting to the ED, and both the pregnancy and delivery were uneventful. She experienced a left-sided headache 5 days after delivery and noted discomfort behind her left ear 9 days after delivery. The patient had a history of migraine and surgical treatment for recurring left patellar subluxation, but she had not experienced muscle or tendon rupture. She was diagnosed with Scheuermann's kyphosis at around 14 years of age, and previous genetic screening for drug metabolism revealed a heterozygous variant of the methylenetetrahydrofolate reductase (*MTHFR*) gene, NM_005957.5:c.677C>T (NP_005948.3:p.Ala222Val; rs1801133). The patient had no other significant medical history—including trauma, smoking, or diagnosis of vascular or connective tissue diseases—and no relatives with a known history of connective tissue disease or stroke. Laboratory results were normal except for a slight decrease in protein S activity.

**Figure 1:**
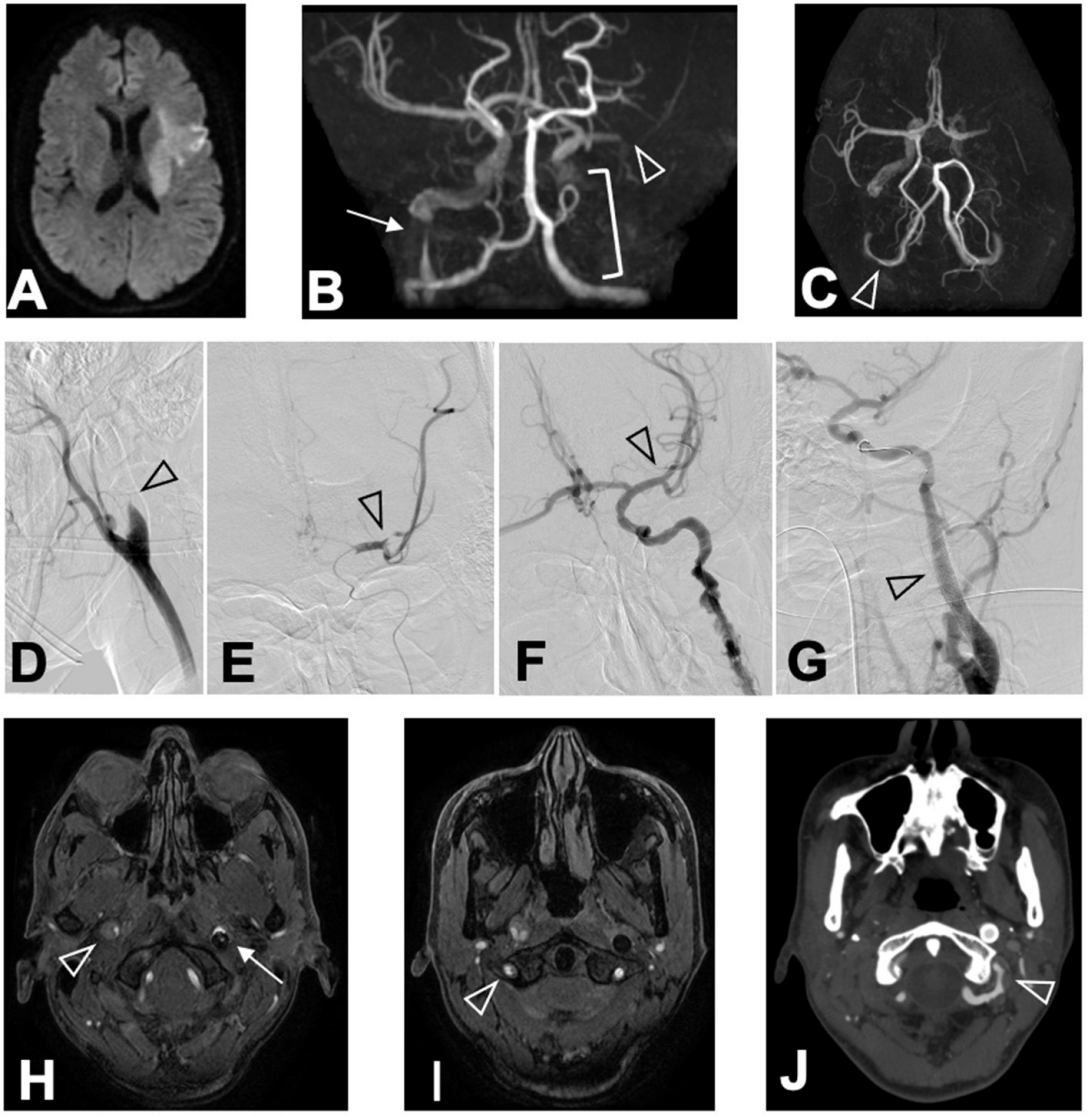
Chronological changes of CCAD lesions. MRI (DWI) on arrival revealed **(A)** hyperintense lesions in the left frontal lobe, the left temporal lobe, and the left corona radiata in DWI sequence. MRA revealed **(B)** M2 segment occlusion of the left MCA (arrowhead), occlusion of the left ICA (bracket), and severe stenosis of the right ICA (arrow) in coronal view, and **(C)** luminal irregularity of the V3 segment of the right VA (arrowhead) in basal view. Angiography conducted at ED revealed **(D)** the left ICA occlusion (arrowhead), **(E)** occlusion of the M2 segment of the left MCA (arrowhead), **(F)** recanalized left MCA after thrombectomy (arrowhead), and **(G)** stented left ICA after angioplasty (arrowhead). MRA performed 24 h after hospitalization revealed **(H)** an intimal flap in the right ICA (arrowhead), the stented left ICA (arrow), and **(I)** intimal flap in the right VA (arrowhead). CTA performed 15 days after hospitalization revealed **(J)** luminal irregularity of the V3 segment of the left VA (arrowhead).

Computed tomography (CT) of the head showed no brain hemorrhage. On diffusion-weighted imaging, magnetic resonance imaging (MRI) revealed hyperintense lesions in the left frontal and temporal lobes and left corona radiata, but fluid-attenuated inversion recovery sequences showed no parenchymal hyperintensity; the Diffusion-Weighted Imaging-Alberta Stroke Program Early Computed Tomography Score was 7 (Figure 1A). Magnetic resonance angiography (MRA) and endovascular angiography revealed occlusion of the M2 segment of the left middle cerebral artery (MCA), occlusion of the left internal carotid artery (ICA), severe stenosis of the right ICA, and luminal irregularity of the right vertebral artery (VA; Figures 1B–E,H,I). Both the anterior communicating artery and the left posterior communicating artery remained patent.

Administration of rt-PA was started 105 min after symptom onset, and mechanical thrombectomy *via* the occluded left ICA restored the left MCA perfusion to grade 3 of the Thrombolysis in Cerebral Infarction scale 185 min after symptom onset (Figure 1F). Angioplasty and stenting were performed on the left ICA to avoid re-occlusion (Figure 1G), while we conservatively observed the severe stenosis of the distal portion of the right ICA. No abnormalities were identified in the left VA at this time. Whole-body CT after rt-PA administration and thrombectomy revealed no hemorrhagic complications in the brain or the uterus.

The patient's NIHSS score improved to 9 immediately after thrombectomy and 3 12 h after symptom onset. The patient received dual antiplatelet therapy (aspirin and clopidogrel). We added antithrombotic therapy (argatroban) briefly until the absence of the hypercoagulable state was confirmed. The patient was clinically monitored for potential hemorrhagic complications with CT and MRI.

Despite the normal appearance of the left VA on the initial investigation and CT angiography (CTA) performed 8 days after patient hospitalization, dissection of the V3 segment of the left VA with stenosis was identified by CTA 15 days after hospitalization (Figure 1J). There was no restenosis of the left ICA stent, and both the true and false lumens of the right ICA and right VA remained patent. The patient did not develop any new symptoms during this time.

The patient was discharged from the hospital 25 days after admission. She noted a deterioration in the dexterity of her right leg, which was unidentifiable by objective neurological examination. Nine weeks after discharge, CTA confirmed false lumen occlusion of the left VA at 78 days post-identification.

The patient underwent screening for underlying connective tissue diseases. Her fasting serum methionine concentration (20.4 nmol/ml) was within the normal range, and her homocysteine concentration (4.8 nmol/ml) was slightly below normal. Genetic screening identified a heterozygous *COL5A1* gene variant, NM_000093.4:c.4943A>G (NP_000084.3:p.Asp1648Gly; rs746071518). There were no known or potentially pathogenic variants among the other 44 connective tissue disease-associated genes, including *COL3A1, COL5A2, TGFBR1/2, SMAD3, TGFB2/3, FBN1, ACTA2, MYH11*, and *FLNA* (Supplementary Table 1).

**Figure 2:**
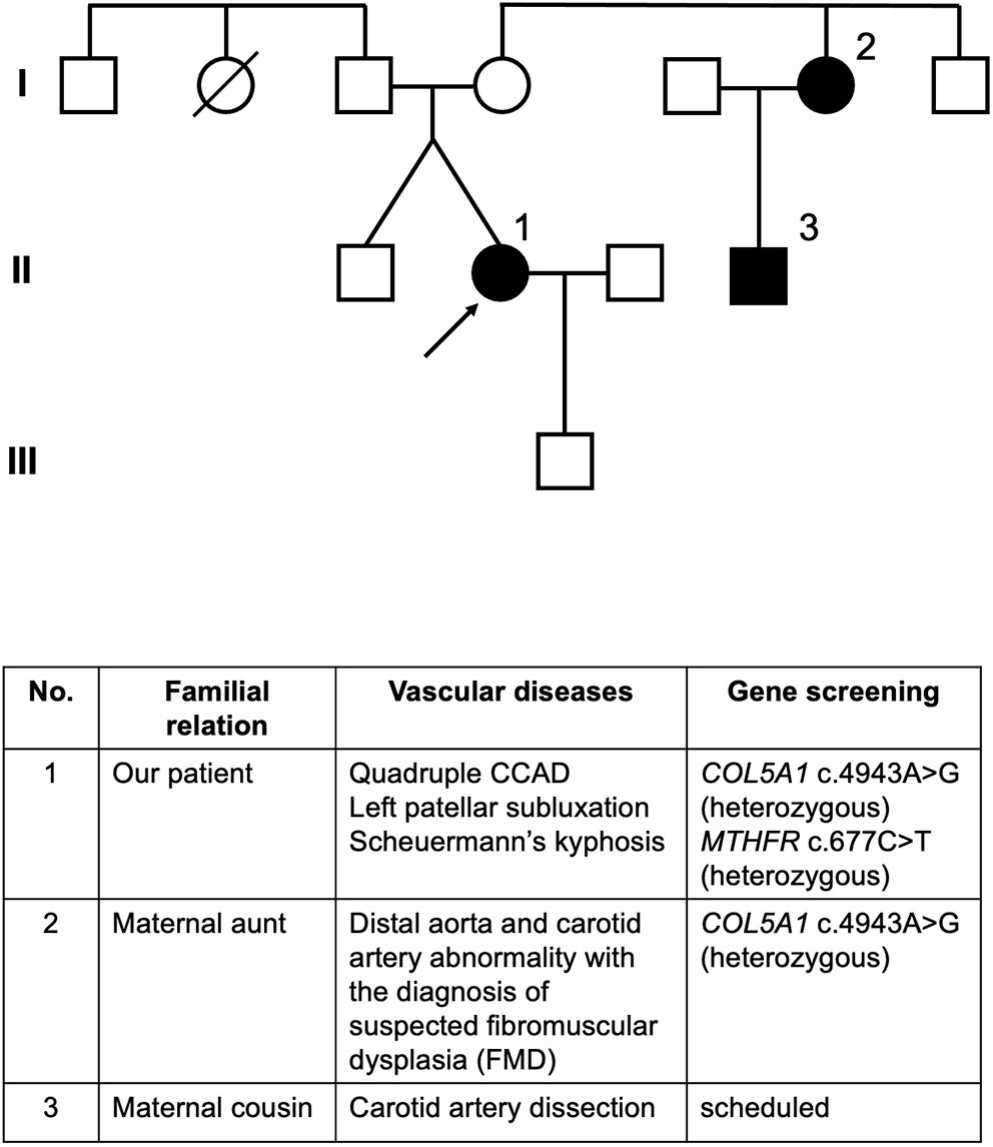
Pedigree of the patient. Individuals affected with arterial lesions are colored in black. Circle 1 indicated by an arrow is our patient. Circle 2 indicates the maternal aunt of our patient who developed abnormalities in the distal aorta and carotid artery with the diagnosis of suspected FMD, whose genetic screening also identified heterozygous *COL5A1* c.4943A>G mutation. Square 3 indicates the maternal male cousin of our patient who developed carotid artery dissection and was scheduled for gene screening.

Following the hospitalization of the patient, her two genetically related relatives developed arterial disorders (Figure 2). Her maternal aunt was diagnosed with suspected fibromuscular dysplasia (FMD) involving the distal aorta and carotid artery and tested positive for the same heterozygous gene variant as our patient. The maternal cousin of the patient was diagnosed with spontaneous carotid artery dissection without stroke after suffering severe headaches, and he was scheduled for genetic screening. The mother of the patient is suspected to be a carrier of the gene variant, but she has not been diagnosed with vascular events or connective tissue diseases. The patient remained free of sequelae at the 4-year follow-up period, and no novel arterial abnormalities were detected on the annual MRA.

## Discussion

All four lesions in our patient revealed chronological changes consistent with acute arterial dissections. The left ICA dissection presumably occurred 9 days after the delivery, which probably led to artery-to-artery embolism resulting in infarction in the left MCA region. Cerebral circulation at the ED arrival was maintained mainly by the left VA and the right posterior and anterior communicating arteries. This increased hemodynamic burden might have triggered the left VA dissection.

The *COL5A1* is an autosomal gene encoding the alpha-1 chain of type V collagen; its pathogenic variants are associated with Ehlers-Danlos syndrome types I and II (12). The *COL5A1* gene variant c.4943A>G identified in our patient results in the substitution of acidic aspartic acid by neutral glycine at codon 1,648, and its clinical significance remains unelucidated (13). The substitution is not located in the triple helical region of the processed type V collagen. Still, it potentially affects the C-terminal propeptide and the protein processing. There are reports of other potentially pathogenic variants in the C-terminal propeptide region of the type V collagen (14, 15). This variant has a very low frequency among non-Finnish Europeans (gnomAD, 0.007%) and the overall population (gnomAD, 0.003%) without reported cases of *COL5A1*-related disease (16).

Other presumed predispositions to the extensive CCAD presentation include migraines (17) and a history of recurring left patellar subluxation and Scheuermann's kyphosis. Meanwhile, the uncomplicated vaginal delivery with epidural analgesia suggested that excessive vessel wall stress during labor is unlikely pathogenesis. Marfan syndrome, FMD, segmental arterial mediolysis, alpha-1 antitrypsin deficiency, polycystic kidney disease, and other vasculitis were ruled out. The previously identified *MTHFR* gene variant is assumed to be clinically insignificant, considering the common prevalence of *MTHFR* C677T polymorphism among Caucasians, the heterozygous genotype, and the absence of hyperhomocysteinemia. The contribution of *MTHFR* C677T polymorphism to CCAD remains controversial, but previous studies have found no significant association, even in patients with homozygous TT genotype (4, 18–20). The synergistic effect between the *COL5A1* gene variant and the *MTHFR* single nucleotide polymorphisms of our patient remains unknown.

The extensive clinical presentation of our patient and the prevalence of arterial abnormalities in her family warrant further assessment of the pathogenicity of *COL5A1* gene variant c.4943A>G, which may help identify populations at risk of spontaneous arterial dissections.

## Data Availability Statement

The original contributions presented in the study are included in the article/Supplementary Material, further inquiries can be directed to the corresponding authors.

## Ethics Statement

Ethical review and approval was not required for the study on human participants in accordance with the local legislation and institutional requirements. The patients/participants provided their written informed consent to participate in this study. Verbal informed consent was obtained from the individual(s) for the publication of any potentially identifiable images or data included in this article.

## Author Contributions

TI, TO, NS, OI, and YO treated the patient and wrote the manuscript. SA, HN, AS, MI, KY, NK, HI, and GY treated the patient and revised the manuscript. KT and HM conducted a genetic screening and revised the manuscript. TU contributed to the discussion of radiological findings and revised the manuscript. All authors contributed to the article and approved the submitted version.

## Conflict of Interest

The authors declare that the research was conducted in the absence of any commercial or financial relationships that could be construed as a potential conflict of interest.

## Publisher's Note

All claims expressed in this article are solely those of the authors and do not necessarily represent those of their affiliated organizations, or those of the publisher, the editors and the reviewers. Any product that may be evaluated in this article, or claim that may be made by its manufacturer, is not guaranteed or endorsed by the publisher.
