# Supplementary material for: Case Report: Spontaneous Postpartum Quadruple Cervicocephalic Arterial Dissection With a Heterozygous COL5A1 Variant of Unknown Significance
Source: Front Neurol. 2022 Jul 12;13:928803. doi: 10.3389/fneur.2022.928803 (PMC9327320; doi:10.3389/fneur.2022.928803)
Supplement: Supplementary file 1 [file Table_1.DOCX]

Supplementary Material

**Supplementary Table 1.**

**List of the 45 connective tissue disease-associated genes screened for mutations.**

| Gene | Transcript ID |
| --- | --- |
| *ACTA2* | NM_001613.2 |
| *ACVRL1* | NM_000020.2 |
| *ADAMTSL4* | NM_019032.5 |
| *B3GALT6* | NM_080605.3 |
| *B4GALT7* | NM_007255.2 |
| *BGN* | NM_001711.5 |
| *BMPR2* | NM_001204.6 |
| *CHST14* | NM_130468.3 |
| *COL1A1* | NM_000088.3 |
| *COL1A2* | NM_000089.3 |
| *COL3A1* | NM_000090.3 |
| *COL5A1* | NM_000093.4 |
| *COL5A2* | NM_000393.3 |
| *DSE* | NM_001080976.2 |
| *EFEMP2* | NM_016938.4 |
| *ELN* | NM_000501.3 |
| *ENG* | NM_000118.3 |
| *FBLN5* | NM_006329.3 |
| *FBN1* | NM_000138.4 |
| *FBN2* | NM_001999.3 |
| *FKBP14* | NM_017946.3 |
| *FLNA* | NM_001456.3 |
| *FOXE3* | NM_012186.2 |
| *HCN4* | NM_005477.2 |
| *LOX* | NM_002317.6 |
| *LTBP4* | NM_001042544.1 |
| *LTBP4* | NM_001042544.1 |
| *MYH11* | NM_002474.2 |
| *MYLK* | NM_053025.3 |
| *NOTCH1* | NM_017617.4 |
| *PLOD1* | NM_000302.3 |
| *PMEPA1* | NM_020182.4 |
| *PRKG1* | NM_001098512.2 |
| *SKI* | NM_003036.3 |
| *SLC2A10* | NM_030777.3 |
| *SLC39A13* | NM_152264.4 |
| *SMAD2* | NM_005901.5 |
| *SMAD3* | NM_005902.3 |
| *SMAD4* | NM_005359.5 |
| *SMAD9* | NM_001127217.2 |
| *TGFB2* | NM_003238.3 |
| *TGFB3* | NM_003239.3 |
| *TGFBR1* | NM_004612.3 |
| *TGFBR2* | NM_003242.5 |
| *TNXB* | NM_019105.6 |

The coding region of each gene was screened for mutations; the noncoding region was not tested. Whole exome sequencing was not performed in the patient.

**
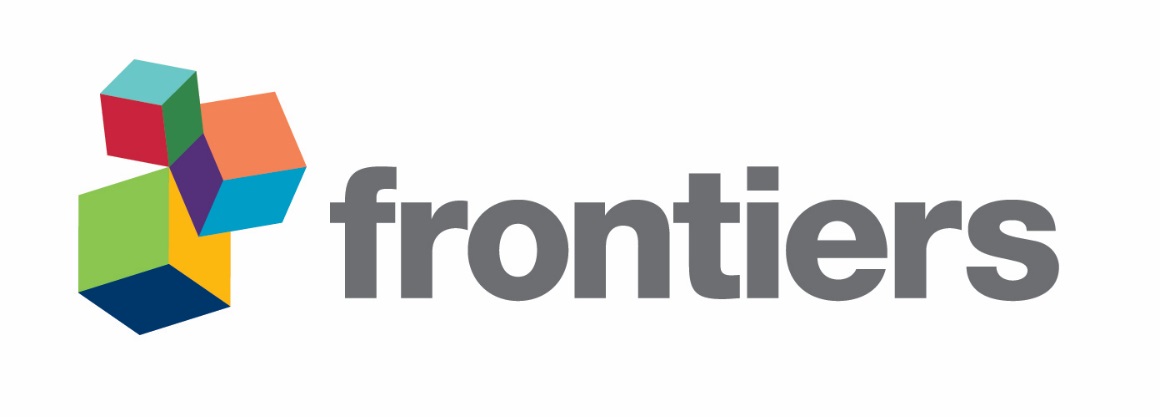
**
